# Response of Various Conduit Arteries in Tachycardia- and Volume Overload-Induced Heart Failure

**DOI:** 10.1371/journal.pone.0101645

**Published:** 2014-08-15

**Authors:** Xiao Lu, Zhen-Du Zhang, Xiaomei Guo, Jenny Susana Choy, Junrong Yang, Mark Svendsen, Ghassan Kassab

**Affiliations:** 1 Department of Biomedical Engineering, Indiana University Purdue University Indianapolis, Indianapolis, Indiana, United States of America; 2 Department of Surgery, Indiana University Purdue University Indianapolis, Indianapolis, Indiana, United States of America; 3 Cellular and Integrative Physiology, Indiana University Purdue University Indianapolis, Indianapolis, Indiana, United States of America; Loyola University Chicago, United States of America

## Abstract

Although hemodynamics changes occur in heart failure (HF) and generally influence vascular function, it is not clear whether various HF models will affect the conduit vessels differentially or whether local hemodynamic forces or systemic factors are more important determinants of vascular response in HF. Here, we studied the hemodynamic changes in tachycardia or volume-overload HF swine model (created by either high rate pacing or distal abdominal aortic-vena cava fistula, respectively) on carotid, femoral, and renal arteries function and molecular expression. The ejection fraction was reduced by 50% or 30% in tachycardia or volume-overload model in four weeks, respectively. The LV end diastolic volume was increased from 65±22 to 115±78 ml in tachycardia and 67±19 to 148±68 ml in volume-overload model. Flow reversal was observed in diastolic phase in carotid artery of both models and femoral artery in volume-overload model. The endothelial function was also significantly impaired in carotid and renal arteries of tachycardia and volume-overload animals. The endothelial dysfunction was observed in femoral artery of volume-overload animals but not tachycardia animals. The adrenergic receptor-dependent contractility decreased in carotid and femoral arteries of tachycardia animals. The protein expressions of NADPH oxidase subunits increased in the three arteries and both animal models while expression of MnSOD decreased in carotid artery of tachycardia and volume-overload model. In conclusion, different HF models lead to variable arterial hemodynamic changes but similar vascular and molecular expression changes that reflect the role of both local hemodynamics as well as systemic changes in HF.

## Introduction

Heart failure (HF) is one of the leading causes of cardiovascular morbidity and mortality in Western countries. HF is accompanied by alteration of hemodynamic conditions, which is due to the triggers of complex reflex changes in the sympathetic, endocrine, and rennin systems [1]–[3]. HF is also associated with subcellular abnormalities that are associated with cardiac hypertrophy and vascular dysfunctions.

A critical effect of HF is reduced blood flow in the cardiovascular system resulting from mild to severe reduction in cardiac output (CO). The reduction of CO in HF is usually accompanied by low ejection fraction (EF) and consequently reduced wall shear stress (WSS). In these patients, the heart usually beats faster to compensate for compromised EF. This often leads to transient retrograde flow and negative WSS in arteries during each cardiac cycle. The alteration of WSS may cause endothelial cell dysfunctions [4] which likely contributes to vascular pathophysiology of HF including increased total peripheral resistance which in turn affects the heart through increase in afterload. Endothelial dysfunction can be due to increased reactive oxygen species (ROS) generation and reduced nitric oxide (NO) bioavailability. The specific mechanisms responsible for the interplay between hemodynamic (low and reverse flows) and vascular dysfunction in HF remain relatively unknown. Although increased oxidative stress with reduced nitric oxide (NO) bioavailability has been proposed as a potential mechanism in HF [5], it is unclear which factors are primary in the initiation and progression of vascular dysfunction in HF. It is also not clear whether the etiology of HF affects the conduit arteries differentially.

The objective of present study is to understand whether the specific etiology of HF affects the conduit vessels with varying distance from the heart differentially despite a similar end stage of heart function. An additional objective was to determine whether local or systemic factors are more important determinants of vascular function in animal models of tachycardia and volume overload-induced HF. Accordingly, we studied cardiac function of tachycardia and volume-overload animals and associated hemodynamic parameters in carotid, femoral, and renal arteries. The vascular function and proteins expression of eNOS and NADPH oxidase of various arteries were determined to assess the various biomarkers of blood vessels in the two different HF models.

## Materials and Methods

Domestic swine (55 to 65 kg) were provided by Michigan State University and housed at Indiana University School of Medicine Facilities (Laboratory Animal Resource Center). The pigs had ad libitum access to water and food. A room temperature of 20–22°C and humidity of 30% to 70% were maintained. The animals were carefully checked for preexisting diseases and acclimated for at least 3 days before undergoing the surgical procedure. All animal experiments were performed in accordance with national and local ethical guidelines, including the Principles of Laboratory Animal Care, the Guide for the Care and Use of Laboratory Animals and the National Society for Medical Research, and an approved Indiana University School of Medicine IACUC protocol (Permit Number:3051) regarding the use of animals in research.

### Tachycardia Model

Six animals were used in a pacing-induced tachycardia model. The pigs were fasted overnight and surgical anesthesia was induced with TKX (Telazol 10 mg/kg, Ketamine 5 mg/kg, Xylazine 5 mg/kg) and maintained with isoflurane 1–2%. Ventilation was provided with a respirator to maintain physiological P_CO2_ and P_O2_ at approximately 35 and 100 mmHg, respectively. Electrocardiographic (ECG) leads were attached to the animal limbs and cardiac electrical signals were monitored on a Physio-Control Lifepak 9P defibrillator. Body temperature was maintained at 37°C to 38°C and pH at 7.4±0.1. The anesthesia was monitored and recorded once per every 10 minutes during the procedure. The adequacy of anesthesia was confirmed by stability of respiration and heart rate, absence of palpebral reflex and jaw tone, and no limb withdrawal reflex.

A thoracotomy was performed along the fourth intercostal space and the chest cavity was opened to fully expose the heart. Two platinum pacemaker electrodes (Medtronic, Minneapolis, MN) were placed on the surface in the lateral LV free wall at 0.5 cm apart and secured by suture. The pace maker was placed in a percutaneous pocket near the neck for easy access. The function of the pace maker was tested and the pacing threshold was determined. Pacing was started one or two weeks after the animals were completely recovered from surgery. The initial pacing rate was set at 210 beats/min for one week followed by 190 beats/min for underwent additional three weeks as described earlier [6]. Five animals served as sham which received open-chest surgery without implantation of a pace-maker.

### Volume-overload Model

Seven animals were anesthetized similar to those described above. An incision was made in the mid abdomen and the aorta and vena cava were exposed near the common iliac artery. The adventitia of the aorta and vena cave was removed over an area twice as long and twice as wide as the arteriotomy. Both aorta and adjacent vena cava were partially occluded with tissue clamps. An incision (1 cm long) was made on vena cava wall on the occluded side to open the cava cavity. From the inside of the vena cava cavity, another small incision was made on vena cava wall and through the adjacent, occluded aorta wall. A 2.5 mm aortic punch (Medtronic, Minneapolis, MN) was then used to create the aortic-vena cava (A-V) fistula at diameter of 6 mm. The free edges of the fistula of cava and aorta was sutured together (7/0 suture), to maintain the incision open (A-V fistula). The first incision made on vena cava was closed with 7/0 suture. The animal was then heparinized (100 U/kg) and the clamp released. The patency of the fistula was ensured after 20 minutes of observations and the abdomen was subsequently closed layer by layer. The animals were given Plavix (75 mg/day) and Asprin (81 mg/day) with food daily to prevent clotting and maintain an open fistula. Five animals served as sham that underwent the open-abdominal surgery and the dissection of abdominal aorta and vena cava without the A-V fistula.

### Echocardiography and blood flow measurement

Echocardiography (Philips Ultrasound, Bothell, WA) was performed weekly to evaluate the heart function. Animals were maintained under light anesthesia with 1–2% Isoflurane and 100% oxygen by mask. The animals were then placed on sling for echocardiography. For tachycardia HF animals, the pace maker was turned off for 30 min to attain stable conditions during imaging. After the echocardiographic measurements, the pace maker was turned back on.

In the terminal study, the animals were placed under general anesthesia after the echocardiography procedure described above. The pace maker was turned off and blood sample was collected. One of each carotid, femoral, and renal arteries was carefully exposed. A perivascular ultrasonic flow probe (Transonic Systems Inc. Ithaca, NY) was placed around the middle portion of each vessel segment. The incisions were approximated and the vessels were allowed to recover for at least 30 min. The blood flow of these vessels was then recorded for an additional 30 min. After flow measurements, the vessels were excised for analysis of proteins expression and vascular function.

### In vitro isovolumic myography

The functions of endothelial and smooth muscle cells in vessels from control or paced animals were tested using an isovolumic myograph [7]. In these experiments, the vessel contraction was maintained under isovolumic conditions during the test, and the changes of lumen pressure represented the degree of contraction or dilation of the vessels. The details of method for the isovolumic myograph and testing protocol have been described previously [7]. Briefly, the vessel segments were quickly removed from animals before termination and placed in 4°C saline. The surrounding connective tissues were carefully removed under a microscope and all branches of the vessel were ligated. The vessel was then transferred into the myograph bath chamber filled with physiological salt solution (PSS: in mmol/L: 142 NaCl, 4.7 KCl, 2.7 Sodium HEPES, 3 HEPES acid, 1.17 MgSO_4_, 2.79 CaCl_2_, 5.5 Glucose) and cannulated at two ends to tubes containing PSS. The vessel was stretched to its in vivo length and equilibrated for 40 min with intravascular pressure set at 10 mmHg while the chamber temperature was gradually increased to 37°C. The intravascular pressure was increased to 80% of physiological pressure during the testing. Phenylephrine (PE) and acetylcholine (ACh) dose-response contraction and dilation experiments were performed on each vessel, respectively. The overall contractility of the vessel segment was tested with 60 mM KCl introduced into the chamber. The endothelium-independent vasorelaxation in response to sodium nitroprusside was performed to verify whether vascular smooth muscle (VSM) developed resistance to nitric oxide.

### Western blotting analysis

Stored artery segments were homogenized in lysis buffer containing 50 mM glycerophosphate, 100 µM sodium orthovanadate, 2 mM magnesium chloride, 1 mM EGTA, 0.5% Triton X-100, 1 mM DL-dithiothreitol, 20 µM pepstatin, 20 µM leupeptin, 0.1 U/ml aprotinin, and 1 mM phenylmethylsulfonyl fluoride and then incubated on ice for 1 hr. The sample was centrifuged at 1,000 *g* for 15 min at 1°C, and the supernatant was collected. The total protein was measured by a BCA kit (Bio-Rad). To examine each protein expression, equal amounts of protein (50 µg) from each sample were loaded on each lane and electrophoresed in 4–20% Tris-Glycine gel (Invitrogen) and then transferred onto a polyvinylidene difluoride membrane (Millipore). After being incubated for 2 hrs in 6% dried milk in TBS-Tween buffer, the membrane was incubated overnight at 4°C with specific primary antibody in blocking buffer (anti-p47^phox^ and anti-gp91^phox^, Santa Cruz Biotech at 1∶200 dilution, MnSOD 1∶1000 dilution, Enzo; eNOS 1∶300 dilution, BD). The membrane was then rinsed and incubated with horseradish peroxidase-conjugated secondary antibody (Santa Cruz Biotechnology) for 2 hrs at 1∶5,000 dilutions in blocking buffer. All samples from each group were also probed with anti-GAPDH antibody (1∶5000, Abcam) to correct for sample loading.

### ELISA analysis

Blood samples were centrifuged and plasma was collected and stored in -80°C until ELISA analysis. The 96-well plates precoated with angiotensin II specific antibody were used to measure Angiotensin II in plasma (Abcam's Angiotensin II Human *in vitro* ELISA kit). Standards or test samples were added to the wells of the plates and subsequently an angiotensin II specific biotinylated detection antibody was added and then followed by washing with wash buffer. Streptavidin-Peroxidase Complex was added and unbound conjugates were washed away with buffer. TMB was then used to visualize Streptavidin-Peroxidase enzymatic reaction. TMB was catalyzed by Streptavidin-Peroxidase to produce a blue color product that changed into yellow after adding acidic stop solution. The density of yellow coloration was directly proportional to the amount of angiotensin II captured in plate.

### Data Analysis

All data were expressed as mean±standard deviation (SD). Students' t-test was used to detect differences between pairwise groups. Variance analysis (One-way ANOVA) with post-hoc (Bonferroni) tests was used in time course and Two-way ANOVA with post-hoc (Tukey's) test was used in dose-dependent comparisons. P<0.05 was considered statistically significant.

## Results

In the tachycardia and volume-overload induced HF, the ratios of heart weight to body weight were increased significantly in the 4 week duration in comparison with sham animals (Table 1). This increase occurred in both left and right ventricles but predominately in right ventricle (Table 1). In tachycardia induced HF model, all animals tolerated the rapid pacing and 50% of the animals had abdominal ascites (500–3000 ml) by the end of 4 weeks. In volume-overload induced HF model, the animals developed edema, respiratory distress, and ascites (700–3,700 mml) in 4 weeks. We also found that the level of Angiotensin II in plasma increased in both tachycardia and volume-overload groups in comparison with sham (Table 1).

**Table 1:** Ratio of heart weight to body weight and plasma angiotensin II in sham, tachycardia (Tachy) and volume-overload (VOL) HF animals.

	Sham	Tachy	VOL	P value
Heart/body (g/kg)	2.7±0.02	4.2±0.62	4.1±0.5	<0.05
RV/LV (g/g)	0.32±0.03	0.5±0.06	0.5±0.16	<0.05
LV/BW (g/kg)	2.0±0.03	2.8±0.35	2.7±0.17	<0.05
RV/BW (g/kg)	0.65±0.05	1.4±0.3	1.4±0.43	<0.05
Ang II (ng/ml)	0.09±0.08	0.343±0.219	0.263±0.176	<0.05

P value is the comparison of tachycardia and volume-overload groups with sham. RV: right ventricle. LV: left ventricle. BW: body weight Ang II: angiotensin II.

**Figure 1:**
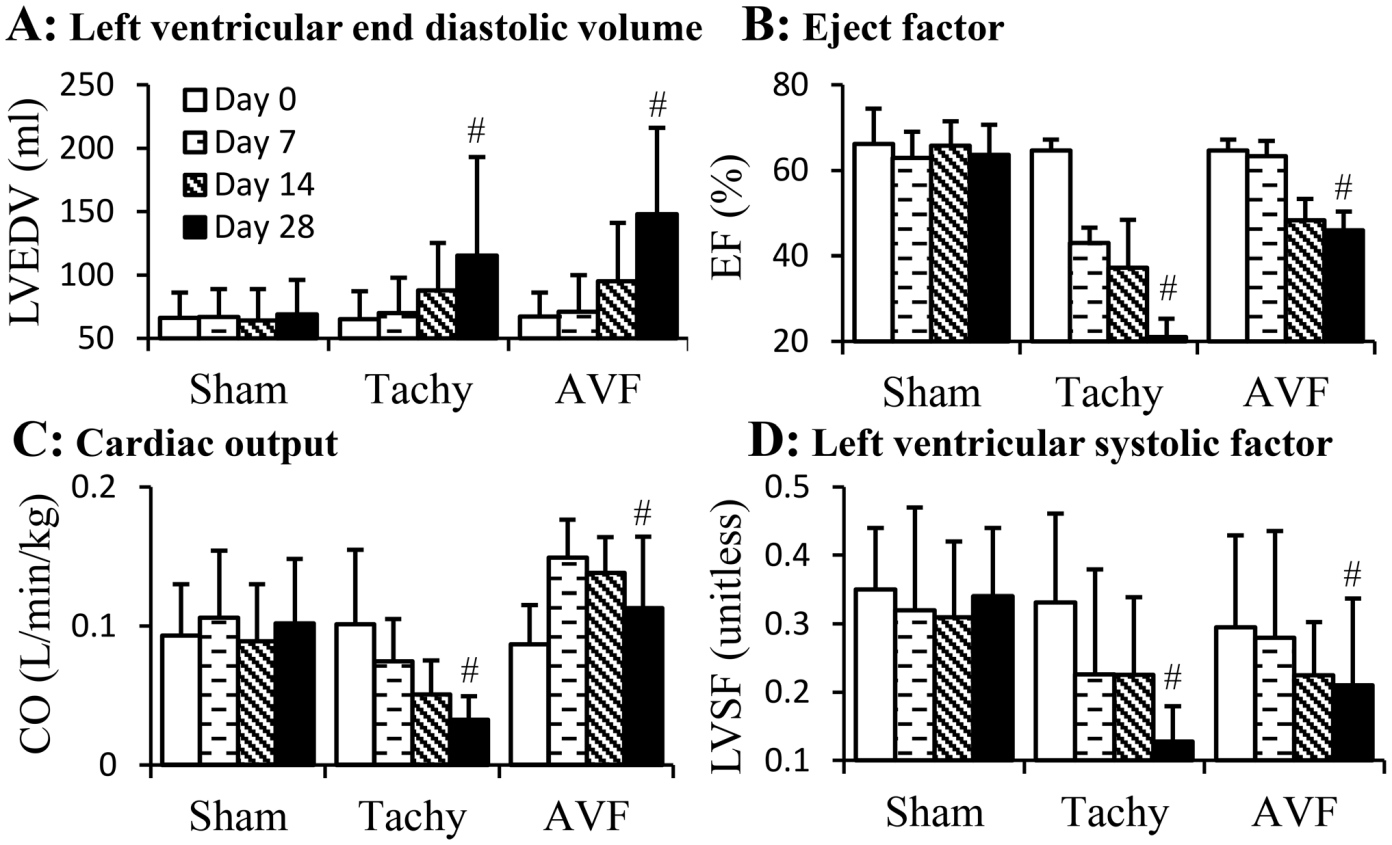
Cardiac parameters of tachycardia and volume-overload models over the four weeks duration. A: left ventricular end diastolic volume (LVEDV); B: ejection fraction (EF); C: cardiac output (CO); D: left ventricular systolic factor (LVSF). Sham, sham group; Tachy, tachycardia group; VOL, volume-overload group. #: p<0.05, One-way ANOVA analysis with post-hoc (Bonferroni) tests to verify the variation of cardiac parameters in time course.

The parameters of cardiac function from echocardiography are represented in Figure 1. The left ventricular end diastolic volume (LVEDV) significantly increased (p<0.05) in both tachycardia and volume-overload HF (Figure 1A). The ejection fraction (EF) decreased significantly (p<0.05) in both tachycardia and volume-overload induced HF (Figure 1B). The cardiac output (CO) decreased monotonically in tachycardia animals but increased in the first postoperative week and decreased in following weeks of volume-overload animals (Figure 1C). The left ventricular systolic factor (LVSF) also decreased (p<0.05) in both tachycardia and volume overload induced HF (Figure 1D).

**Figure 2:**
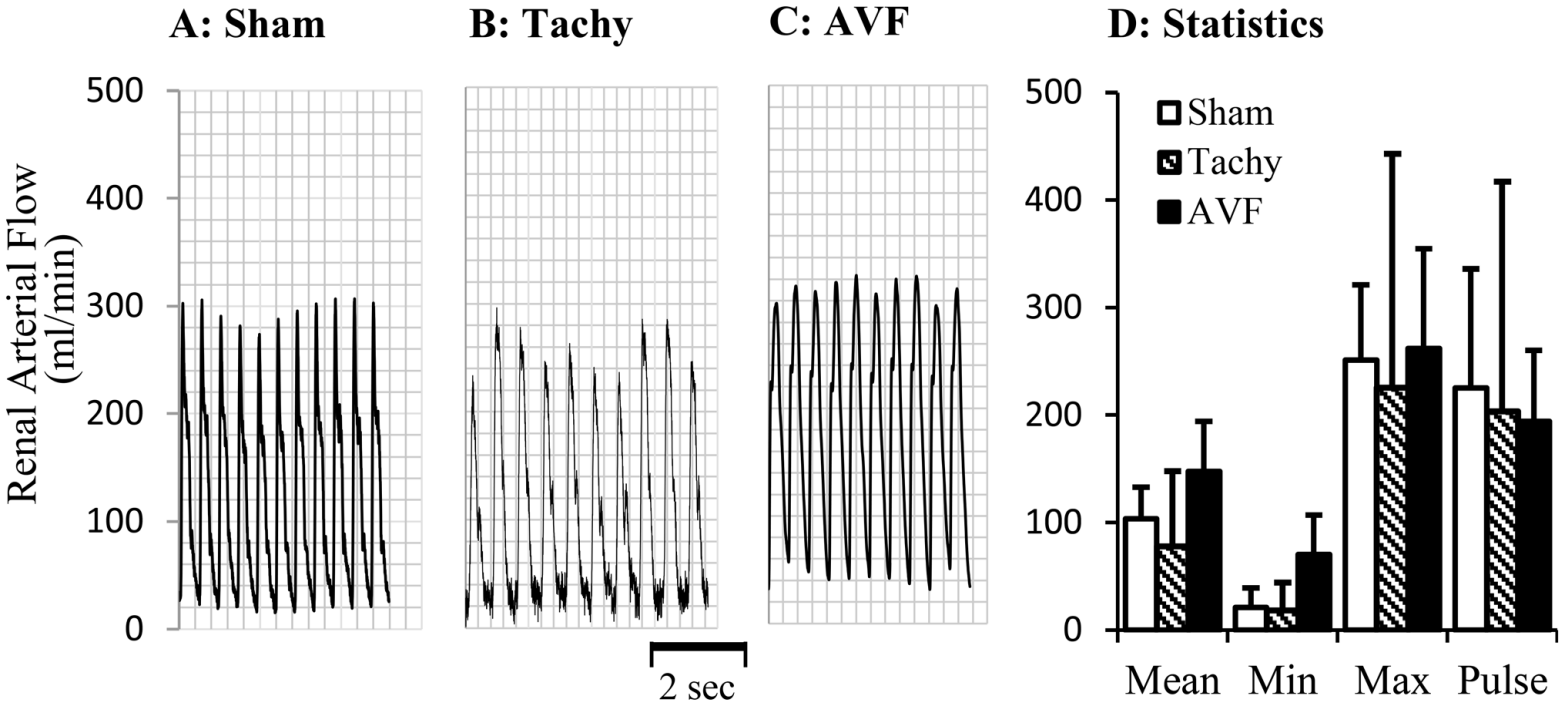
Blood flow in renal arteries at day 28. A representative tracing of blood flow inform A: sham animal; B: tachycardia animal; C: volume-overload animal. D: Statistical data (mean±SD) of blood flow at day 28. Pulse flow is defined maximal flow to minus minimal flow. Sham, sham group; Tachy, tachycardia group; VOL, volume-overload group. *: p<0.05, Student's t-test in comparison with sham.

**Figure 3:**
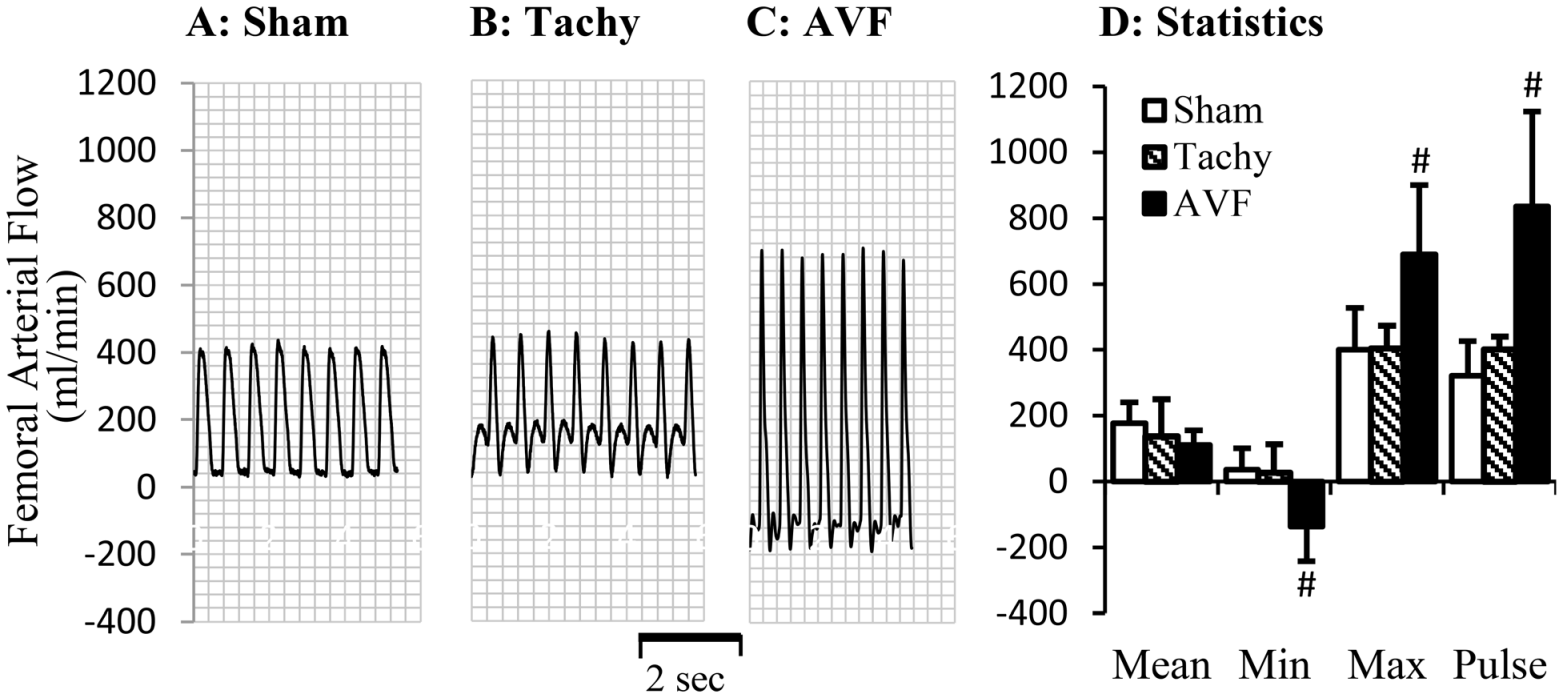
Blood flow in femoral artery at day 28. A representative tracing of blood flow inform A: sham animal; B: tachycardia animal; C: volume-overload animal. D: Statistical data (mean±SD) of blood flow at day 28. Pulse flow is defined maximal flow to minus minimal flow. Sham, sham group; Tachy, tachycardia group; VOL, volume-overload group. *: p<0.05, Student's t-test in comparison with sham.

**Figure 4:**
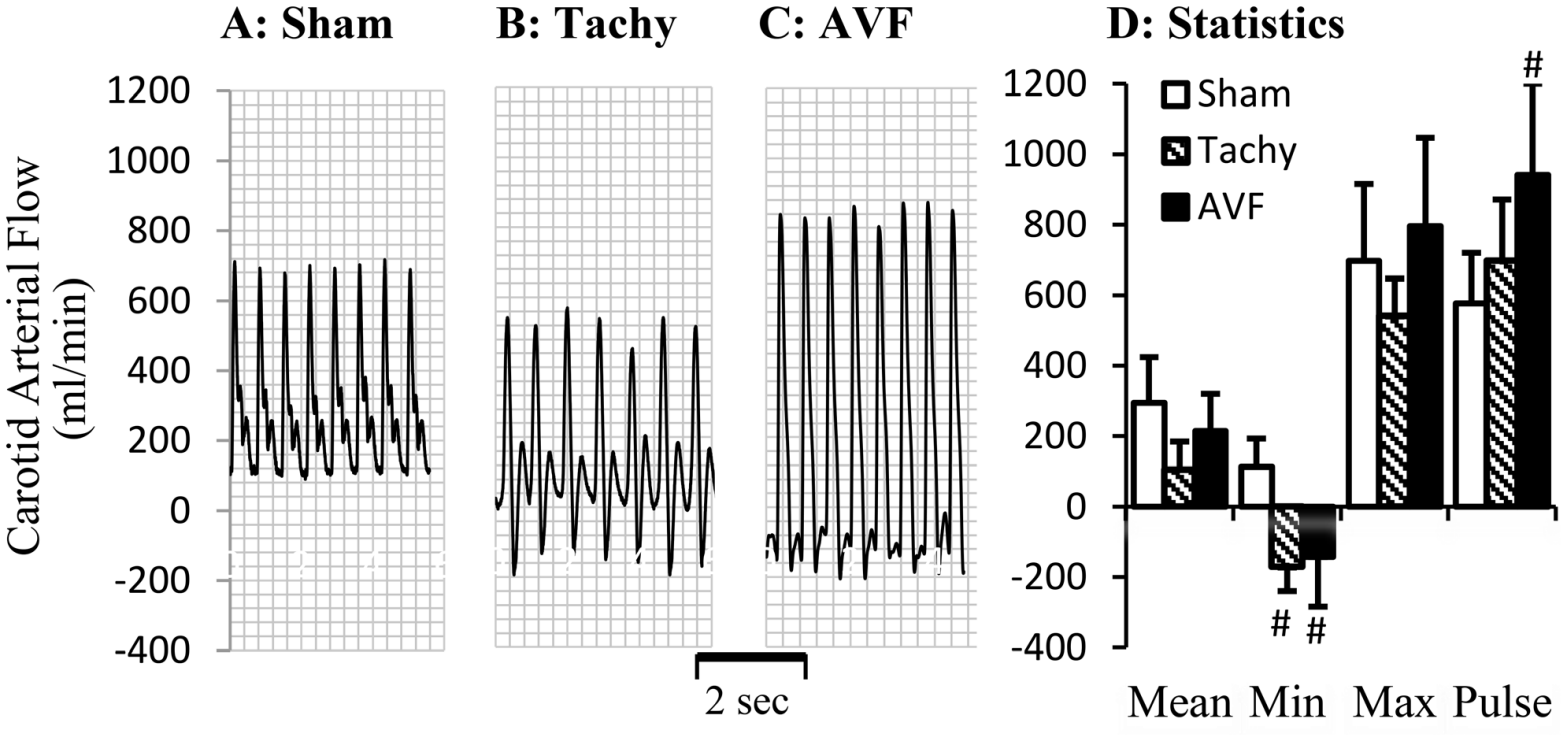
Blood flow measured in carotid artery at day 28. A representative tracing of blood flow from A: sham animal; B: tachycardia animal; C: volume-overload animal. D: Statistical data (mean±SD) of blood flow at day 28. Pulse flow is defined maximal flow to minus minimal flow. Sham, sham group; Tachy, tachycardia group; VOL, volume-overload group. *: p<0.05, Student's t-test in comparison with sham.

The blood flows in various arteries at the time of acute studies are represented in Figures 2–4. The mean blood flow and pulse flow (maximal minus minimal flow) in renal artery did not change statistically in both tachycardia (pace maker was turned off during blood flow measurements) and volume-overload animals (Figure 2). In femoral artery, there was no significant flow reversal during diastolic phase in sham and tachycardia animals (Figures 3A, B and D). In A-V fistula volume-overload animals, a significant flow reversal during diastolic phase was observed in femoral artery and the pulse flow increased significantly in tachycardia and volume-overload groups (Figures 3C and D). The significant flow reversal was observed during diastolic phase in carotid artery four weeks after tachycardia and volume-overload but not in sham animals (Figures 4A, B and C). The pulse flows in carotid artery in volume-overload animals were significantly increased in comparison with shams (Figure 4D). The mean flow in carotid artery decreased in tachycardia group in comparison with sham.

**Figure 5:**
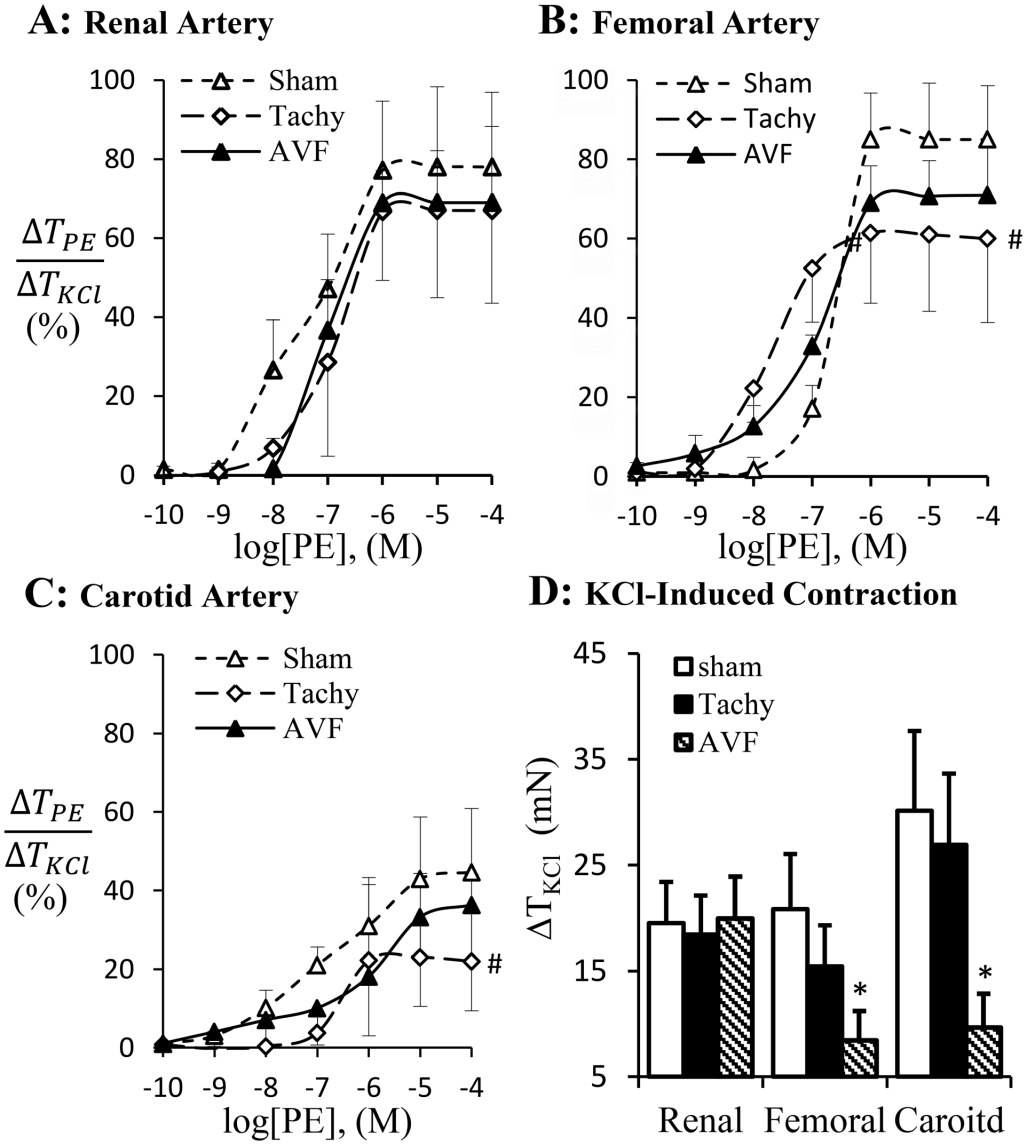
Adrenergic agonist PE-induced arterial contraction was normalized by KCl-induced contraction. A: renal artery contraction; B: femoral artery contraction; C: carotid artery contraction. D: potassium chloride (KCl) induced contraction. Sham, sham group; Tachy, tachycardia group; VOL, volume-overload group. #: p<0.05, Two-way ANOVA analysis with post-hoc (Tukey's) test to detect the difference of the dose-dependent groups. *: p<0.05 Students' t-test in comparison with sham.

The phenylephrine-induced vascular contraction is represented in Figure 5. Phenylephrine activates adrenergic receptor to induce vascular smooth muscle contraction which was blunted in femoral and carotid arteries but not in renal artery of tachycardia animals (Figure 5A–C). The adrenergic receptor dependent contraction did not change in volume-overload (Figure 5A–C). KCl–induced receptor-independent contraction was attenuated in femoral and carotid arteries in volume-overload animals but not in renal artery (Figure 5D). Although the receptor-independent vasoconstriction did not change statistically in the carotid and femoral arteries in tachycardia animals, a decreased tendency was observed (Figure 5D). Based on the contractility analysis, the adrenergic activation of carotid and femoral arteries appears blunted in tachycardia but not in volume-overload animals.

**Figure 6:**
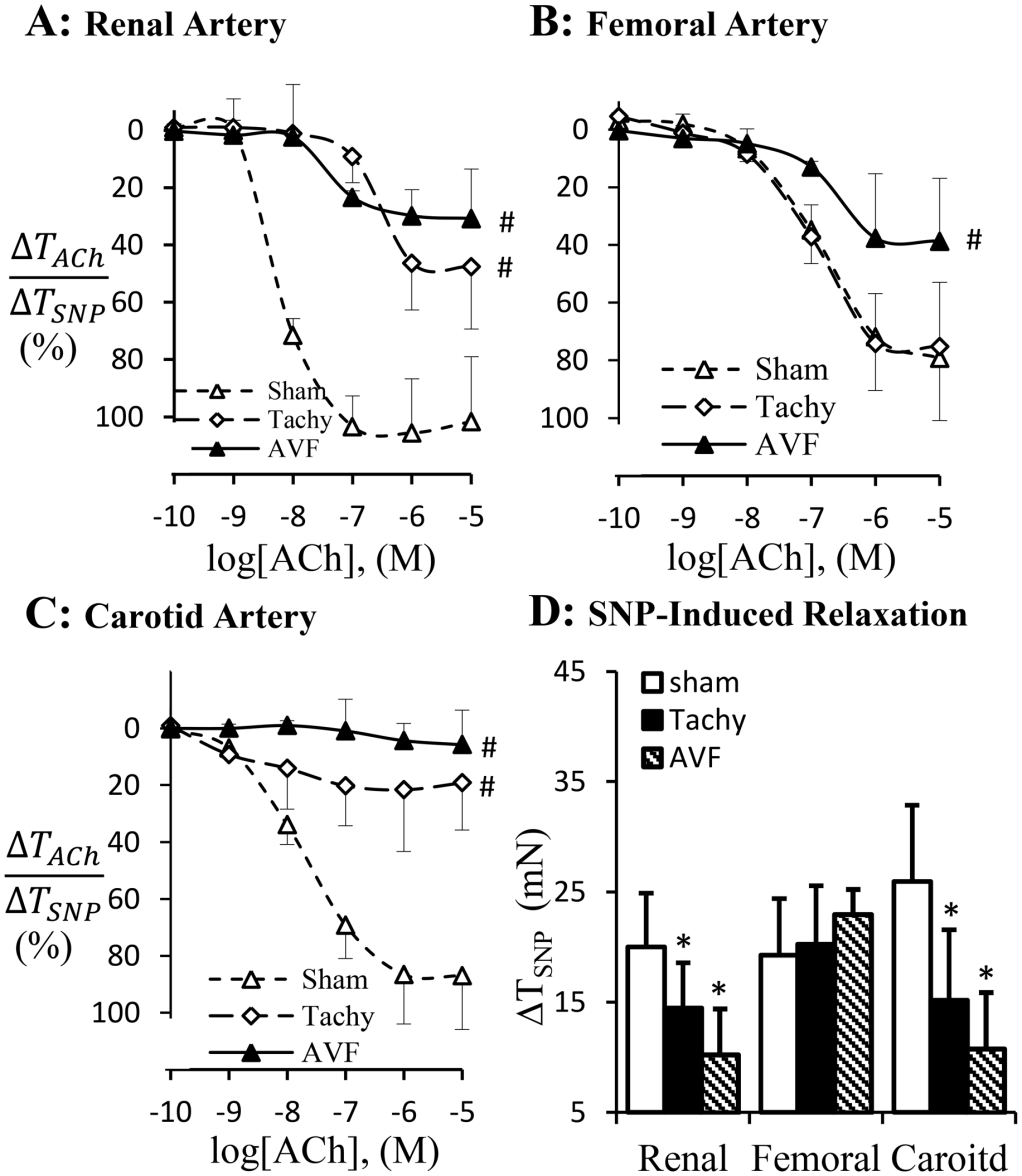
Endothelium-dependent vascular relaxation. Acetylcholine-induced vasodilation was normalized with sodium prusside (SNP, NO donor) induced vasodilation in renal (A), femoral artery (B) and carotid artery (C). D: SNP induced vasodilation. Sham, sham group; Tachy, tachycardia group; VOL, volume-overload group. #: p<0.05, Two-way ANOVA analysis with post-hoc (Tukey's) test to detect the difference of the dose-dependent groups. *: p<0.05 Students' t-test in comparison with sham.

The endothelium-dependent vasodilation in response to acetylcholine (ACh) is represented in Figure 6. The significant attenuation of endothelium-dependent vasodilation was observed in renal and carotid arteries in tachycardia and volume-overload animals (Figure 6A, C). The endothelium-dependent vasodilation of femoral artery was attenuated in volume-overload but not in tachycardia (Figures 6B). The endothelium-independent vasodilation in response to sodium nitroprusside was significantly attenuated in renal and carotid arteries in both tachycardia and volume-overload animals and did not change in femoral artery experimental groups (Figure 6D).

**Figure 7:**
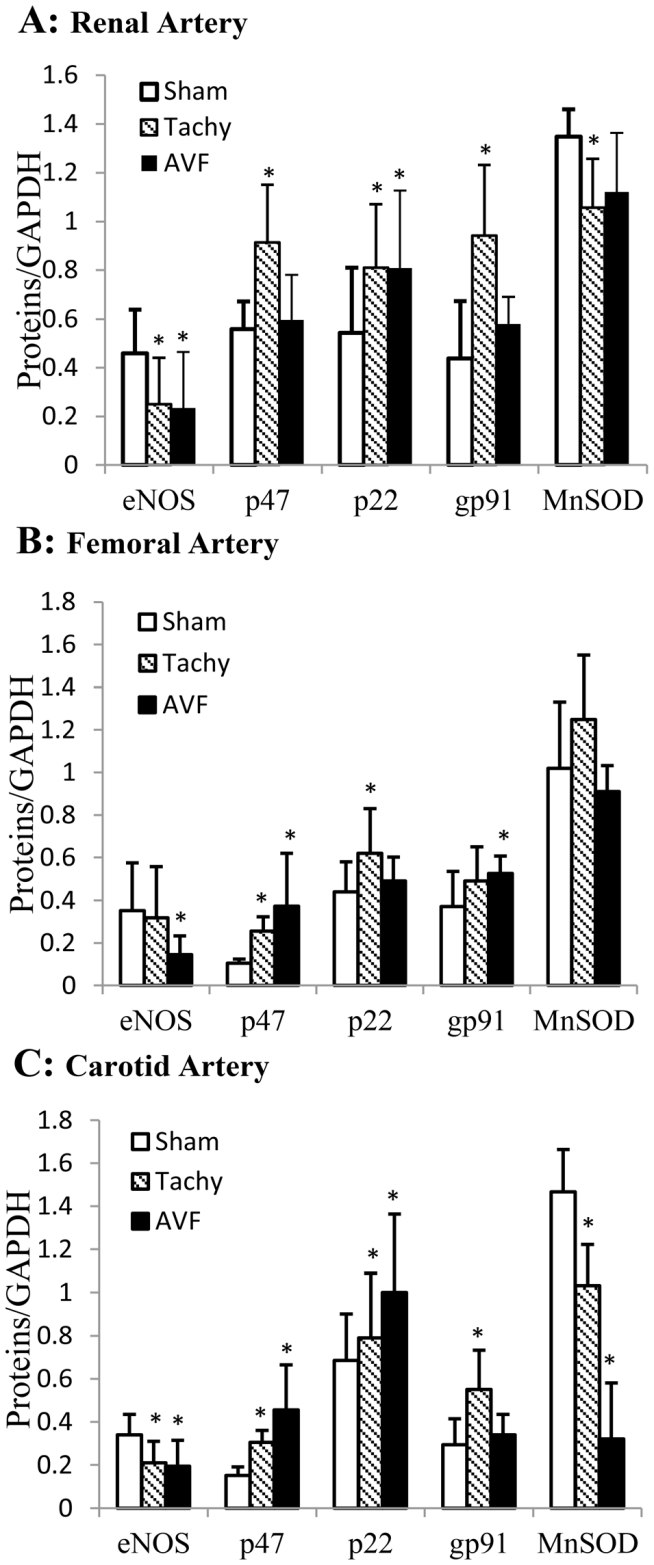
Expression of eNOS, p47^phox^, p22^phox^, gp91^phox^, and MnSOD in various arteries measured by western blotting. A: renal artery; B: femoral artery; C: carotid artery. Equal amount of protein of each sample were used. The results were expressed as ratio of densities of target protein and GAPDH. Sham, sham group; Tachy, tachycardia group; VOL, volume-overload group. *: p<0.05 Students' t-test in comparison with sham.

Expressions of several proteins were determined by Western blot in artery segments collected from sham, tachycardia, and volume-overload animals (Figure 7). The expression of eNOS was down-regulated in renal and carotid arteries of tachycardia model (Figure 7A and C) and all three arteries of volume-overload model (Figure 7). The expression of p47^phox^ was up-regulated in all three arteries of tachycardia model (Figure 7) and femoral and carotid arteries of volume-overload model. The expression of p22^phox^ was up-regulated in renal and femoral arteries of tachycardia model and renal and carotid arteries of volume-overload model (Figure 7). The expression of gp91^phox^ was up-regulated in renal and carotid arteries of tachycardia model and femoral artery of volume-overload model (Figure 7). The MnSOD expression was down-regulated in renal and carotid arteries of tachycardia model and carotid artery of volume-overload model (Figure 7).

The cardiac function and hemodynamic changes and their effects on vascular functions and molecular expression are summarized in Table 2. The cardiac function at four weeks was consistently compromised in tachycardia and volume-overload HF models. The hemodynamic changes were not consistent in the various vessels in the two animal models while the vascular function and molecular expression changes were fairly consistent with the femoral artery being least affected.

**Table 2:** Summary of heart and vascular function, hemodynamic, and biochemical changes at 4 weeks of tachycardia and volume-overload.

	Tachycardia	Tachycardia
LV/BW	**↑**	**↑**
ANG II	**↑**	**↑**
LVEDV	**↑**	**↑**
EF	**↓**	**↓**
CO	**↓**	**↓**
LVSF	**↓**	**↓**

Notes: The functional and biochemical variations are denoted using ↑(increase), ↓ (decrease), No (not found), Yes (found), — (no change), 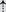 (increased but not statistically, p values were between 0.051 to 0.063), and 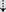 (decreased but not statistically, p values were between 0.052 to 0.061).

## Discussion

In tachycardia and volume-overload HF models, carotid, femoral and renal arteries were exposed to either systemic (Ang. II, plasma oxidative stress, etc.) or systemic plus hemodynamic (flow reversal) factors. We observed that systemic factor alone compromised endothelial function of renal artery and systemic factor plus flow reversal compromised endothelial function of carotid and femoral arteries. The oxidative stress was increased in all three arteries of tachycardia and volume-overload HF models while adrenergic dependent contraction was decreased in femoral and carotid arteries but remained unchanged in renal arteries of both HF models. The observation underscores different response of various blood vessels to systemic effects. These findings suggest that hemodynamics (flow reversal) is an important regulator of endothelial function and receptor-dependent vasoconstriction in addition to the systemic effects in tachycardia and volume-overload HF models.

Tachycardia or volume-overload HF model induced by either pace maker or A-V fistula have been well established in large and small animals [8], [9]. In tachycardia model, the pacing rates vary and the time required to develop HF is different depending on the animal species. We followed a previously used rapid pacing to induce HF in swine with higher rates initially (230 bpm) followed by lower pacing rate at 190 bpm [6]. The tachycardia animals in our experiments showed typical changes such as reduced ventricular function [10]–[12]. As seen in Figure 1, the EF and LVEF were significantly reduced. The hypertrophy of the heart was also observed as shown in Figure 1. The increase of heart weight (Table 1) is predominantly in right ventricle as reported by other groups [12], [13]. These findings confirmed that the animals in our present study developed typical changes in tachycardia HF model. For volume-overload model, the EF decreased from 65% to 45%. As shown in Figure 1, the LVEDV was also doubled suggesting a sever LV dilation as shown previously by other groups [12], [14]. In our experiments, the volume-overload animals developed significant complications including edema, ascites and respiratory distress. Animals would not survive longer to observe further decrease of EF without medical interventions. Therefore, the EF of HF animal with volume-overload in our study was higher than those reported in humans that are under medical treatment. However, the LV dysfunction was similar to those reported previously [15].

In previous studies, the cardiac and vascular functions in HF have been intensively studied. The increased peripheral artery resistance has been attributed to neurohumoral pathways including the rennin-angiotensin and sympathetic nervous system [1], [3]. Our present study suggests that regional blood flow change may also contribute to regulation of vascular function. We have shown previously that acute tachycardia by pacing induced transient hemodynamic changes in medium sized peripheral arteries, including decreased systemic pressure and near-wall retrograde flow or full flow reversal [16]. In tachycardia animals, the flow reversal was found in the carotid artery during diastolic phase but not in the femoral artery. In volume-overload animals, the reversed flow was observed in both femoral and carotid arteries (Figure 3–4). Reverse flow was not observed in renal artery of either HF model. The reason that blood flow reversal is observed in some vessels but not others is because flow pulsatility and transient reversal dependent not only on cardiac hemodynamics (inlet into the vessel) but also the impedance of microcirculation. Although cardiac functions and hemodynamics were affected similarly in the various vessels, the regulation of impedance in various microcirculations is likely very different; e.g., renal microcirculation tone is regulated by various hormones (Ang. II, norepinephrine, etc.) and hence prevents flow reversal. The response of renal artery to various agonists is also different from other peripheral arteries [17]–[18]. Therefore, Ang. II and oxidative stress in this study result in different vascular dysfunction of renal artery from carotid and femoral arteries. The receptor-independent vasoconstriction which reflects the contractility of vascular smooth muscle was also compromised in the carotid artery of tachycardia and volume-overload animals and femoral artery of only volume-overload animals (Figure 5D). The adrenergic receptor dependent contraction in carotid and femoral arteries, however, was attenuated in tachycardia animals but did not change in volume-overload (Figure 5B and C). The decrease in adrenergic receptor dependent contraction in tachycardia model may indicate blunted adrenergic activation. This implies that adrenergic activation may be more relevant to HF models (tachycardia or volume-overload) and overshadows the hemodynamic contribution (reversal flow and pulse flow). Although the contractility differences (e.g., receptor dependent and independent vasoconstriction) were largely noted for all vessels, the renal arteries in both models were not affected (Table 2). Endothelial dysfunction, however, was observed in the renal artery of tachycardia and volume-overload animals despite the lack of flow reversal, which suggests factors other than local hemodynamics factors. Hence, the endothelial function was compromised in the renal artery but the vascular smooth muscle cell function remained preserved at this duration of the model. Although the impaired endothelial function of renal artery may be one of the complications of heart failure [19], the cause and effect relations remain unclear.

The primary source of superoxide in endothelial cells stems from NADPH oxidase. NADPH oxidase which has been extensively studied in phagocyte consists of both membrane components (p22^phox^, gp91^phox^ and Rac1) or catalytic core proteins and cytosolic components (p47^phox^ phagocyte oxidase, p67^phox^ phagocyte oxidase, Rac, and Rho GDI) or regulatory subunits [20]. The endothelial NADPH oxidase (Nox) shares many similarities with those of phagocytes. All the classical NADPH oxidase subunits are expressed in endothelial cells [21]. The level of superoxide in tissue depends on the balance between production and removal. SODs are enzymes that catalyze the dismutase of superoxide and serve as important antioxidant [22]. In the present study, we found that the expression of NADPH oxidase subunits increased in all arteries of tachycardia and volume-overload animals, which approximately correlates somewhat with endothelial dysfunction (Figure 7). The decrease in MnSOD in carotid artery and renal artery of tachycardia animals also correlates with endothelial dysfunction (Figure 7). Several lines of evidence support the hypothesis that superoxide likely mediated vascular dysfunctions in our study. First, Ang II has been shown to increase in HF and induce superoxide production in endothelial cells [23]. In combination with increase Nox subunit (p47^phox^) and decreased antioxidant (SOD), Ang II may play an important role in superoxide production of the three arteries. Second, altered hemodynamics may play a major role. We have previously shown evidence that NADPH oxidase has a directional response to shear stress [24]. In the present study, the retrograded flow was observed in carotid artery from tachycardia and volume-overload animals and femoral artery in volume-overload animals.

In summary, the present study investigated the systemic factors (physio-pathological parameters such as circulating Ang. II and oxidative stress), hemodynamic changes and vascular dysfunctions in renal, carotid and femoral arteries in two HF animal models induced by chronic tachycardia and volume-overload. In these models, systemic factors changed significantly and local reverse blood flow was found in carotid and femoral arteries but not in renal artery. The endothelial dysfunction coincided with arteries having flow reversal (femoral and carotid in volume overload and carotid artery of tachycardia model) but was also found in vessels without flow reversal (renal arteries of both tachycardia and volume overload models). This suggests an important role for systemic factors in renal endothelial dysfunction in comparison with carotid and femoral arteries in which systemic factors plus flow reversal result in endothelial dysfunction. Adrenergic dependent vascular smooth muscle activation was compromised in both femoral and carotid arteries of both HF models but not in renal arteries. The molecular expressions were largely similar in all vessels of both HF models. Although tachycardia and volume-overload HF model showed similar impaired of LV dysfunction at four weeks, there was variability in arterial hemodynamics and the functional and molecular expression reflected both the local hemodynamic as well as systemic changes in HF.
